# A Practical Approach to Condition Assessment of Asphalt-Covered Concrete Bridge Decks on Korean Expressways by Dielectric Constant Measurements Using Air-Coupled GPR

**DOI:** 10.3390/s20092497

**Published:** 2020-04-28

**Authors:** Ji-Young Rhee, Ko-Eun Park, Kang-Hyun Lee, Seong-Hoon Kee

**Affiliations:** 1 Korea Expressway Corporation, Hwaseong 18489, Korea; need@ex.co.kr (J.-Y.R.); kepark@ex.co.kr (K.-E.P.); tunnelslope@ex.co.kr (K.-H.L.); 2 Department of Architectural Engineering, Dong-A University, Busan 49315, Korea

**Keywords:** ground-penetrating radar, dielectric constant, service age, concrete bridge deck, asphalt overlay

## Abstract

The main objectives of this study are to investigate the variations of the dielectric constant of concrete on Korean expressways by using a 1 GHz air-coupled Ground Penetrating Radar (GPR) system and to develop a practical approach to the condition assessment of concrete bridge decks with asphalt overlay on Korean expressways by dielectric constant measurements. A total of 684 GPR investigations of 601 actual concrete bridge decks, which are in service between 2 and 43 years, were carried out during the period between 1999 and 2013. Statistical analysis revealed that the dielectric constant of asphalt-covered concrete bridge decks reduced with service age and this trend continued until service age of over 40 years. As a result, this study provides a practical dielectric constant curve that could be used for condition evaluation of top concrete in asphalt-covered bridge decks with consideration of concrete age. Based on regression analyses of the GPR field survey data and experiences through the field survey, a double cut-off dielectric constant criterion was proposed for condition assessment of asphalt-covered concrete bridge decks on Korean expressways. In addition, a GPR field survey was performed at an actual bridge on the Yeongdong expressway in Korea to test the proposed GPR signal interpretation method. The field survey results provide fundamental data to better understand the variation of the dielectric constant of concrete in actual bridges with asphalt overlay and to develop a practical approach to condition assessment of asphalt-covered concrete bridge decks on Korean expressways by dielectric constant measurements using air-coupled GPR.

## 1. Introduction

Expressway bridges are essential elements to connect road segments on various terrain conditions and to constitute the expressway as a single network. It has been reported that the physical life of a bridge could extend to over 100 years when cared with by a systematic maintenance and management program (e.g., Brooklyn Bridge, constructed in 1883, 137 years old, and Williamsburg Bridge, constructed in 1903, 117 years old). In Korea, the service life of a concrete structure was first defined in 2004 [1]. It categorizes structures into three grades according to importance and specified service life of structures. However, a recent study in Korea Expressway Corporation (KEC) says that the average service life of reconstructed bridges in Korean expressways was about 30 years, which is only at the level of the lowest target service life. It was reported that the main reason for the reconstruction of bridges was the deficiency of concrete decks [2]. From the perspective of a road engineer, it is of great importance to evaluate the current condition of concrete bridge decks, and if necessary, to make the right decision for appropriate maintenance actions, which will keep the concrete bridge decks in sound condition and enhance the service life of bridges.

Infrastructure management agencies in many countries have used Ground Penetrating Radar (GPR) as a tool for evaluating concrete bridge decks because of its capability of visualizing the sub-surface condition of asphalt-overlaid concrete bridge decks and rapid operation at a traffic speed. American Society for Testing and Materials (ASTM) first established the standard test method for condition assessment of bridge decks using GPR in 1997 [3]. Several research teams in the US have successfully used GPR technology for condition assessment of bridge decks [4,5,6,7,8]. In Europe, GPR technology has been widely used as a rapid condition assessment tool for civil infrastructure systems [9,10,11]. Recently, more than 300 experts from 28 countries have gathered to promote the use of GPR in the field of civil engineering [12]. In Korea, KEC has implemented the use of GPR equipment in the late 1990s to evaluate the surface and/or near-surface of bridge decks in Korean expressway networks. The GPR technology in Korea has been actively and continuously utilized as a rapid non-destructive evaluation tool for condition assessment of concrete bridge decks until now. 

In Korea, the evaluation of concrete bridge decks with asphalts overlay is mainly based on the relative permittivity (or dielectric constant) of the top concrete of bridge decks [13,14]. Good and/or damaged concrete bridge decks were surveyed by a 1 GHz air-coupled GPR system. Deteriorated concrete in the wet conditions, with high-water content, results in a high value of concrete dielectric constant. The high-water content in deteriorated concrete could result in further degradation associated with freeze-thaw action and acceleration of concrete degradation from various sources (chloride penetration, corrosion of reinforcing bars, etc.). A test region with a relative permittivity value of 12 or above has been interpreted as a deteriorated (or potentially deteriorated) area [13,14]. Generally, the single cut-off dielectric constant criterion of a GPR signal has resulted in good correlation with the actual deterioration of concrete bridge decks in the relatively wet conditions in the field. However, it may not be suitable under very dry conditions since the voids caused by deterioration (e.g., enhanced porosity, microcracks, delamination, etc.) would be filled by air, which would tend to lower the overall dielectric constant. Actually, recent reviews on an intensive GPR field survey by KEC showed that the single cut-off relative permittivity criterion is too simplified to take into account the effect of various influencing factors (age of concrete, void ratio and moisture content of concrete) on the relative permittivity of concrete in actual concrete bridge decks. For example, the single cut-off criterion could not effectively identify deteriorated concrete associated with enhanced porosity and/or distributed surface-breaking cracks in the relatively dry conditions. Therefore, it is of importance to better understand the variations of relative permittivity of concrete with various influential factors for more reliable condition assessment of concrete bridge decks using relative permittivity measurements. 

There have been many previous studies investigating the effects of influencing factors on the variation of relative permittivity of concrete. Zhang et al. [15] found out that the relative permittivity of cement paste rapidly dropped from the time of casting to 30 h of early aging. Robert [16] also observed the rapid decrement in the relative permittivity of concrete up to 300 days of age, and explained this observation in relation to cement hydration. Rhim and Büyüköztürk [17] investigated the variation of the relative permittivity of hardened concrete specimens in various saturation conditions in a frequency range of 0.1 to 20 GHz. In the study, they observed that the relative permittivity of concrete specimens in the wet conditions was almost twice the value of the specimens in the oven-dry conditions. Rhim and Jeong [18] measured the relative permittivity and attenuation factors of mortar and concrete specimens in various moisture contents. Lai et al. [19] examined the change of relative permittivity of concrete mixed with light and ordinary aggregates at 90 days of age. However, most experimental data were obtained in the laboratory and did not effectively consider the actual field effects, such as seasonal variations and age-related changes over the long-term. Recently, Rhee et al. [20] investigated the effect of some influencing parameters (concrete ages and variations of relative humidity of air) on the relative permittivity of concrete decks in actual bridges on Korean expressways with ages from dozens of days to 27 years. The study was only focused on bare concrete decks with limited numbers of bridges (53 bridges on Korean expressways).

The primary objectives of this study are to investigate the variations of relative permittivity of concrete under asphalt-covered concrete bridge decks on Korean expressways by using a 1 GHz air-coupled Ground Penetrating Radar (GPR) and, based on statistical analyses of long-term GPR field survey data, to develop a practical GPR signal interpretation method for reliable condition assessment of actual concrete bridge decks with asphalt overlay on Korea expressways. For these purposes, this study carried out the following four main tasks: (1) investigation on influencing factors of concrete relative permittivity, using GPR equipment, for asphalt-covered bridge decks in Korean expressways that are in service from 1999 to 2013, (2) establishment of a practical formula describing the age-related variations of concrete relative permittivity, (3) proposal of a practical GPR signal interpretation method for condition assessment of asphalt covered concrete bridge decks based on the practical relative permittivity formula and double cut-off relative permittivity criterion and (4) test of the practical GPR signal interpretation method by applying to an actual bridge on the Yeongdong expressway in Korea. The results of this research would provide fundamental data to better understand the variation of relative permittivity of concrete in actual bridges on Korean expressways and to develop a practical GPR signal interpretation method for more reliable condition assessment of concrete bridge decks with asphalt overlay.

## 2. Background: Relative Permittivity of Concrete

The electromagnetic properties of a material can be generally expressed in terms of complex permittivity and complex permeability. Concrete is a dielectric and nonmetallic material, and most dielectric materials are nonmagnetic. Since permeability of a nonmagnetic material is almost the same as the permeability in a vacuum ($4\pi \times 10^{-7}\;H/m$), its electromagnetic property can be expressed as in Equation (1) in consideration of permittivity only.

(1) $$\varepsilon^{*}=\varepsilon -j\varepsilon^{\prime }$$

where $\varepsilon^{*}$ is complex permittivity (F/m), ε and $\varepsilon^{\prime }$ are real and imaginary parts of complex permittivity, respectively, and j is $\sqrt{-1}$. Furthermore, dividing both sides of the equation by the permittivity in vacuum, $\varepsilon_{0}$ = $8.854\times 10^{-12}$ Farad/meter, gives relative permittivity in Equation (2).

(2) $$\varepsilon_{r}^{*}=\varepsilon_{r}-j\varepsilon_{r}^{\prime }$$

where $\varepsilon_{r}^{*}$ is relative complex permittivity, $\varepsilon_{r}$ and $\varepsilon_{r}^{\prime }$ are real and imaginary parts of relative permittivity, respectively. 

The real part of the relative complex permittivity (or dielectric constant) indicates the capability of a dielectric material to accumulate energy from outside the electric field. It has a value greater than one in most cases of solid or liquid state. Moreover, the imaginary part (or loss factor) indicates the energy loss of a dielectric material against outside the electric field and generally has a value much smaller than the dielectric constant. For relatively dry and low conductivity materials, this will be much less than one, and the loss factor can then be ignored. In this study, the relative permittivity of concrete is simply referred to as the real part of the relative complex permittivity (or dielectric constant) ε_r_. 

The propagation velocity of an electromagnetic (EM) wave *V*_m_ in a material can be calculated by dividing the EM wave velocity in air *C*_air_ about 300 mm/ns by the square root of relative permittivity value as follows [21]:

(3) $$V_{m}=\frac{C_{\mathrm{air}}}{\sqrt{\varepsilon_{r}}}$$

Subsequently, the depth of a reflector *d* in a material is calculated from the following equation

(4) $$d=\frac{C_{\mathrm{air}}T}{2\sqrt{\varepsilon_{r}}}$$

where *T* is the two-way travel time (TWTT) of an EM wave in a material. Therefore, a reliable dielectric constant value of a material is necessary to accurately assess the condition of the targets embedded in the media. 

**Figure 1:**
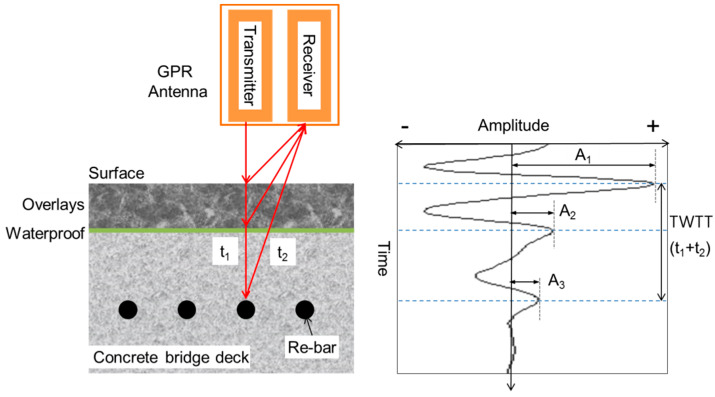
Illustration of Ground Penetrating Radar (GPR) scanning on an asphalt-covered concrete bridge deck and a possible path of electromagnetic (EM) waves.

Figure 1 shows an illustration of the GPR survey for the condition assessment of concrete bridge decks with asphalt overlay. A transmitter in the GPR antenna generates transient EM waves that propagate into the concrete bridge decks under asphalt pavement. Some of the incident EM waves in asphalt pavement are reflected from the AC (Asphalt/Concrete) interface and some transmit through the AC interface. The traditional method for determining the dielectric constant of a medium is the back calculation of the value by using reference drilled cores. This method is still the most common, especially when using ground-coupled GPR systems. The other very popular method is the surface reflection method [22], which can be used with air-coupled GPR systems like the one used in this study. This method uses the reflected amplitude from the bridge deck surface to compare it with the reflection on the metal plate representing a total reflector. By calculating the amplitudes, it is possible to calculate the dielectric constant of both layers (asphalts overlay and concrete deck). Equations (5) and (6) can be used to calculate the dielectric constant values of the first surface (surface of asphalts overlay) and the second surface (in this case, the top surface of concrete of bridge deck), respectively [10].

(5) $$\sqrt{\varepsilon_{r,a}}=\frac{1+\frac{A_{1}}{A_{p}}}{1-\frac{A_{1}}{A_{p}}}$$

(6) $$\sqrt{\varepsilon_{r,c}}=\sqrt{\varepsilon_{r,a}}\times \left[\frac{1-{\left(\frac{A_{1}}{A_{p}}\right)}^{2}+\left(\frac{A_{2}}{A_{p}}\right)}{1-{\left(\frac{A_{1}}{A_{p}}\right)}^{2}-\left(\frac{A_{2}}{A_{p}}\right)}\right]\;$$

where $\varepsilon_{r,a}$ represents the dielectric constant of asphalt concrete; $\varepsilon_{r,c}$ indicates dielectric constant of concrete; $A_{P}$ means the amplitude of an incident EM wave (in this study, the amplitude of the reflected wave from a metal plate); $A_{1}$ and $A_{2}$ mean the amplitude of the reflected waves from the air-asphalts surface and from the A/C interface, respectively (Figure 1). Note that the dielectric constant values determined using Equations (5) and (6) do not exclude the effect of thickness of asphalt concrete. Furthermore, a few oscillations of the first reflected waves from the asphalt concrete surface could interfere with the second reflected waves from the A/C interface, which could cause some errors in the calculation of concrete dielectric concrete using Equations (5) and (6). However, GPR field survey data from Korean expressways revealed that the variation of the dielectric constant due to the variation of asphalt concrete thickness and the interference between the first and second reflected waves is much smaller than that caused by the damage of concrete [23]. Therefore, it can be said that Equations (5) and (6) are still effective for the condition assessment of damaged concrete in the field practice.

## 3. Overview on GPR Field Survey Program

### 3.1. Bridge Status

A total of 684 GPR surveys were performed from a total of 601 bridges on Korean expressways from 1999 to 2013. This study includes bridges with a wide range of ages from 2 years–43 years at the time of survey. All the bridges had concrete decks with asphalts overlay. Table 1 classifies the bridges by survey year and expressway route. In this study, a bridge was classified separately if concrete decks of interest under survey were constructed in different years. Note that most of the top concrete of bridge decks on Korean expressways under investigation were assumed not to be severely deteriorated. The target maintenance level on bridges was set to at least C grade, which means the deteriorated regions in a bridge should be under 10% of a total surface area of a bridge.

### 3.2. Methods of Survey and Analysis

**Figure 2:**
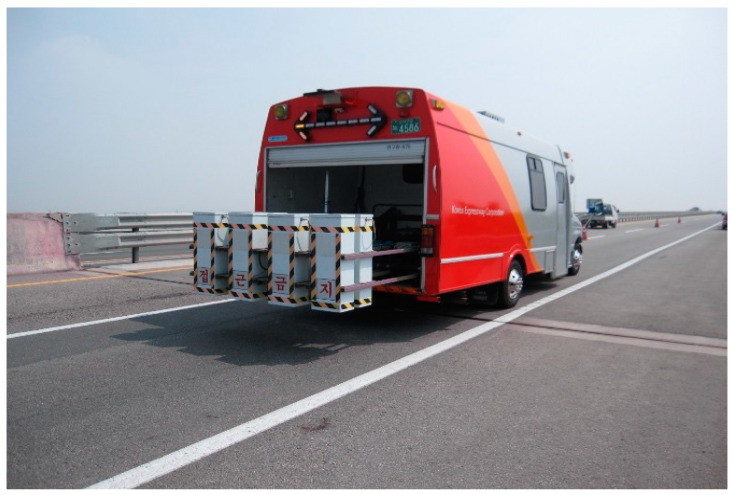
Photo of a GPR survey vehicle using 4-channel 1 GHz air-coupled antennas.

The GPR survey system used was Sir Series of Geophysical Survey Systems Inc. (GSSI) and it used 4-channel 1 GHz air-coupled antennas. The 4-channel antennas were installed at the back of a vehicle that had the distance measuring instrument (DMI) on one of the rear wheels. The GPR system was operated on each traffic lane, including the road shoulder, at the speed of 80–100 km/h for collecting the GPR survey data (Figure 2). The survey was carried out in accordance with the survey protocol of KEC as follows:

- The GPR survey should be conducted at least 24 h after the precipitations,
- The investigation should not be conducted on sunrise or sunset when the relative humidity and moisture on the road change rapidly, and
- Reference GPR signals from a steel plate for GPR signal calibration should be collected immediately before or after GPR surveying.

**Figure 3:**
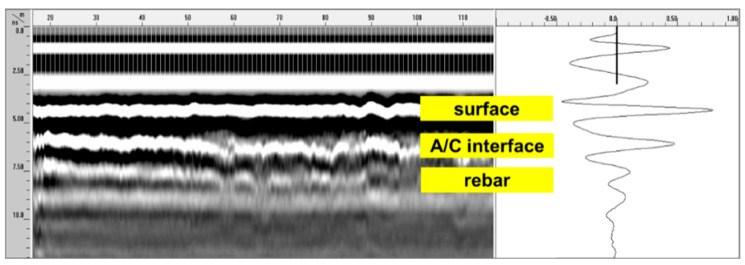
Typical GPR B-scan image on asphalt-covered bridge decks in one of the survey bridges in this study.

Furthermore, the survey was only conducted after confirming that the air-dried surface of an asphalt concrete layer had no standing water on the pavement surface and had no considerable debris that would affect the GPR radar signal interpretations. The GPR signals were acquired with the sampling rate to 12–14 scans/m (i.e., 1 scan for every 70 or 80 mm) in the longitudinal direction. A typical GPR B-scan image obtained on asphalt-covered concrete decks in one of the survey bridges in this study is shown in Figure 3. 

The received signals were stored for the determination of the concrete dielectric constant and location (depth). In this study, a commercially available software, RADAN^®^ [24], was used to analyze the GPR signals. GPR signals collected on the surface of a test bridge were merged into a 3D matrix format (column × row × array). Each 2D array data in the 3D matrix represents a GPR B-scan image (see Figure 3). The vertical axis of the GPR B-scan image represents the two-way-travel time of the reflected GPR signals. The two-way-travel time data were converted to the depth of reflectors in bridge deck components by using Equation (4). Next, the zero-depth was determined at the location where the reference GPR signals were obtained on the steel plate. The adjusted B-scan image visualizes the location of the A/C interface as a strong white line (i.e., positive peaks of the 2nd GPR reflection signals (see Figure 1)). The amplitude values of positive peaks were read from all the GPR traces, that were used to calculate the dielectric constant of the A/C interface. The mean dielectric constant of concrete in each test bridge was evaluated by averaging all the GPR traces obtained from 1000 to 100,000 GPR traces (4 channels × numbers of lane × bridge length (m) × sampling rate (traces/m)). From the raw data set of each test bridge, outliers, which were commonly observed at the bridge joint (steel joint and plain concrete for fixing it about 300–1000 mm width) and with values over 81 (dielectric constant of water), were discarded to compute the average concrete dielectrics and the standard deviation. The distribution of the dielectric constant of each test bridge was assumed to follow the normal distribution since the number of samples for each test bridge is statistically large enough, and each test bridge is in nearly sound condition (expected damaged area is less than 10%). The validity of the normality assumption was verified by performing the Shapiro-Wilk normality test for dielectric constant values measured on the 684 concrete bridge decks considered in this study. It was confirmed that *p*-values of all the bridge decks were greater than 0.05, which indicates the validity of the normality assumption. In this study, the means of concrete dielectric constant were assumed to represent those of concrete decks with asphalt overlay in Korean expressways.

This study used atmospheric relative humidity in order to indirectly investigate the seasonal variation of concrete dielectric constant in asphalt covered concrete bridge decks during the GPR survey. Korea Meteorological Administration (KMA) offers the atmospheric relative humidity data through its website [25]. The atmospheric relative humidity data measured at the meteorological stations near survey bridges on the survey day were used in this study. The detailed meteorological measurement data by the Automatic Weather System (AWS) were used while local weather measurement data measured by the Automated Surface Observing System (ASOS) were used in the absence of such detailed data. 

## 4. Development of a Practical GPR Signal Interpretation Method

### 4.1. Influence of Concrete Age and Relative Humidity of Air 

**Figure 4:**
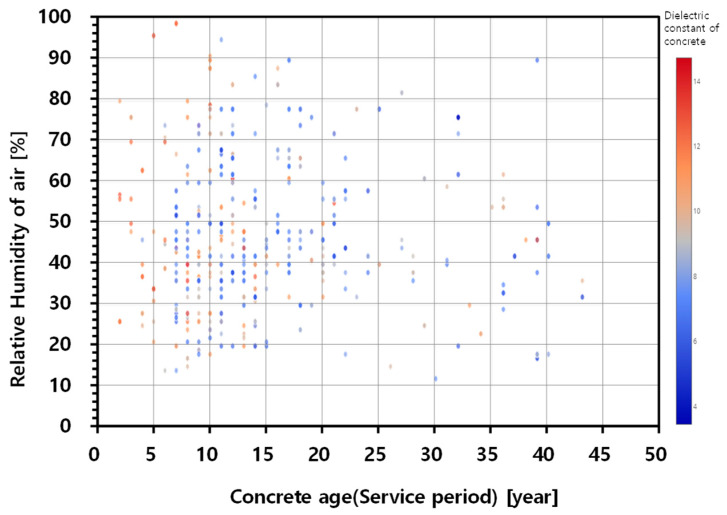
Two-dimensional image representing the distribution of dielectric constant of asphalt-covered bridge decks with relative humidity of air and concrete age (service period).

Figure 4 shows a 2D image representing the variation of the dielectric constant of concrete on the top of bridge decks with asphalt overlay in terms of two main influencing parameters (concrete age and relative humidity of air measured by meteorological stations) [26]. In the figure, the value of the concrete dielectric constant was presented with different colors: higher value with color red and lower value with color blue. In general, the GPR survey data appears to be greatly dispersed, which is mainly due to the variability of bridge components (e.g., concrete age, degree of deterioration, water content, etc.). However, it could be observed that the higher values of relative permittivity are shown in relatively new concrete bridges (0–10 years), which was also observed in concrete bridge decks without asphalt overlay [20]. Young concrete has plenty of water in the concrete pore system, leading to higher dielectric constant than the values in mature concrete in old bridges. 

In this study, an approximate equation relating the dielectric constant of concrete and the two critical parameters (i.e., relative humidity and concrete age) was established by using linear regression to investigate the influence of each variable, as follows,

(7) $$\varepsilon_{r,c}=0.0095\mathrm{RH}-1.065\ln({\mathrm{Age}}_{\mathrm{conc}})+10.92$$

where RH is the relative humidity of the atmosphere (%) and ${\mathrm{Age}}_{\mathrm{conc}}$ is the concrete age at the time of GPR measurements (year). The statistical hypothesis test based on the *t*-test (*p*-values < 0.001) showed that the dielectric constant of concrete is related to each parameter (intercept, RH, and $\ln({\mathrm{Age}}_{\mathrm{conc}})$) at the statistical significance level of 0.01. 

Equation (7) indicates that the relative humidity of air (or the seasonal variation) has only little influence on the dielectric constant of concrete with asphalt overlay. The change of atmospheric relative humidity of 10% results in the change of dielectric constant of top concrete inside the pavement of about 0.11. The ratio of change is only 12% of the case of the bare concrete bridge decks in Korean expressways [20]. Furthermore, the influence of the variation of RH on the dielectric constant of concrete is less than 1% of the variation of $\ln({\mathrm{Age}}_{\mathrm{conc}})$. This is because asphalt concrete and waterproof layer impede circulations of moisture under pavement, which results in different moisture conditions between the pores in top concrete of bridge decks and the air. 

### 4.2. Influence of Concrete Age (Service Performance Period)

**Figure 5:**
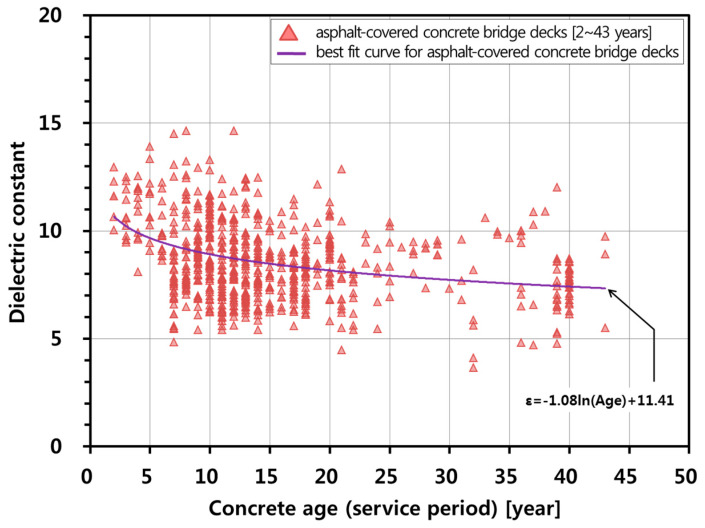
Variation of dielectric constant of concrete in asphalt-covered bridge deck with concrete age (service year).

Figure 5 depicts the variation of the dielectric constant of concrete with concrete age in the survey bridge decks. The dielectric constant of a concrete bridge deck under 10 years of service decreased by about 0.26 a year, while the dielectric constant of concrete over 10 years of service decreased by about 0.03 a year. In other words, as the age increased, the dielectric constant decreased at a reduced rate. This trend followed the behavior observed in the equation for bare concrete bridge deck [20]. In this study, an approximate formula representing the relationship between the dielectric constant of concrete deck with asphalt overlay and concrete age (service period of bridges) was established using nonlinear regression analysis,

(8) $$\varepsilon_{r,c}^{\mathrm{Age}}=-1.08\ln({\mathrm{Age}}_{\mathrm{conc}})+11.41$$

where ${\mathrm{Age}}_{\mathrm{conc}}$ is the concrete age at the time of GPR measurements (year). F-statistics of the nonlinear regression model is 93.590 (*p*-value < 0.001), which means that the relationship in Equation (8) is considered statistically significant at the confidence level greater than 99%. Standard errors of the coefficient of $\ln({\mathrm{Age}}_{\mathrm{conc}})$ and the intercept are 0.111 (*p*-value < 0.001) and 0.296 (*p*-value < 0.001), respectively. Therefore, it can be said that the coefficient of $\ln({\mathrm{Age}}_{\mathrm{conc}})$ and the intercept are statistically significant at the confidence greater than 99%. 

**Figure 6:**
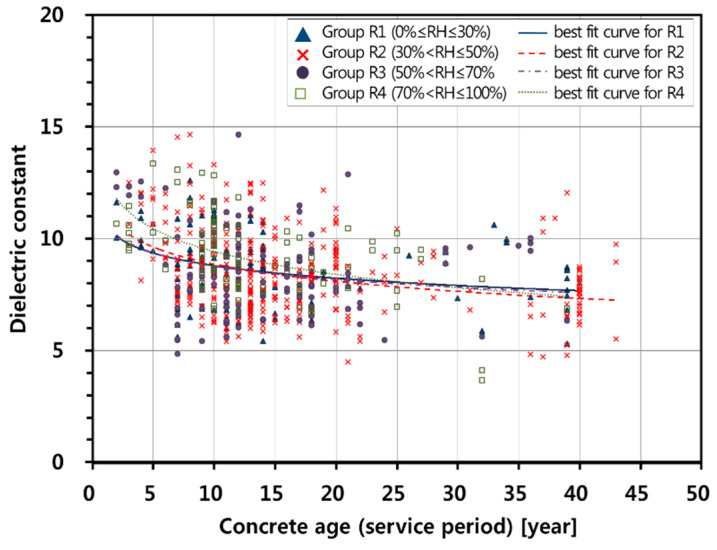
Variation of dielectric constant of asphalt-covered concrete bridge decks in the four bridge groups classified according to relative humidity: RH ≤ 30% (Group R1), 30% < RH ≤ 50% (Group R2), 50% < RH ≤ 70% (Group R3), 70% < RH (Group R4).

**Figure 7:**
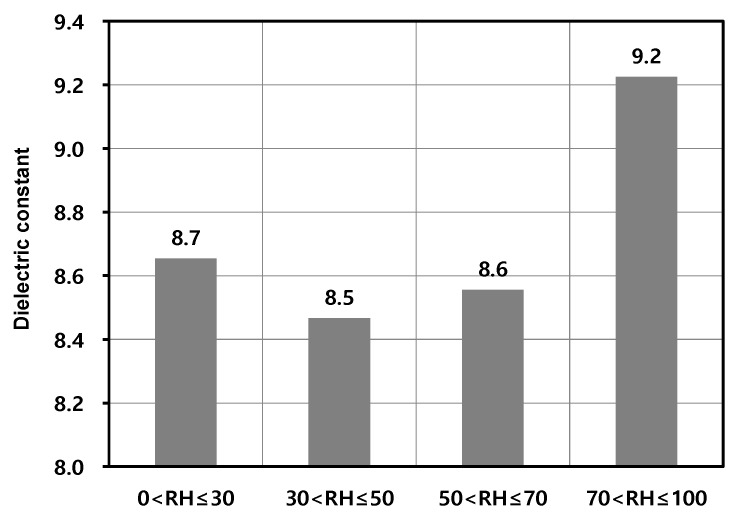
Means of the four groups of bridges classified according to relative humidity: RH ≤30% (Group R1), 30% < RH ≤50% (Group R2), 50% < RH ≤ 70% (Group R3), 70% < RH (Group R4).

In addition, the influence of relative humidity on the relationship between concrete age (service performance period of the bridge) and dielectric constant of concrete was investigated. The research survey bridges were classified into four groups according to relative humidity: RH ≤30% (Group R1), 30% < RH ≤ 50% (Group R2), 50% < RH ≤ 70% (Group R3), 70% < RH (Group R4). Figure 6 shows the variation of the dielectric constant of concrete of asphalt-covered bridge decks in the four groups of bridges classified according to relative humidity. One-way analysis of variance (ANOVA) showed the statistically significant difference between the means of the three groups with RH lower than or equal to 70% (Groups R1, R2, and R3) and the mean of the group with RH greater than 70% (Group R4), with F-value = 4.747 and *p*-value = 0.003 at the significance level of 0.05 (see Figure 7). In addition, it was also confirmed by the analysis of covariance (ANCOVA) that there is a statistically significant difference in the effect of relative humidity on the dielectric constant of Group R4 and the other three groups (Groups R1, R2, and R3), with F-value = 16.446 and *p*-value = 0.00 at the significant level of 0.05. Therefore, it can be said that there is a statistically significant difference between the three groups with RH lower than or equal to 70% (Groups R1, R2, and R3) and the group with RH greater than 70% (Group R4).

The best-fit curve for the relationship between the dielectric constant and age of concrete for the relative humidity less than or equal to 70% was found from nonlinear regression analyses

(9) $$\varepsilon_{r,c}^{\mathrm{Age},\mathrm{RH}\leq 70\%}=-1.01\ln({\mathrm{Age}}_{\mathrm{conc}})+11.41\;\mathrm{for}\;2\leq {\mathrm{Age}}_{\mathrm{conc}}<43\;\mathrm{years}$$

F-statistics of the nonlinear regression model is 63.349 (*p*-value < 0.001), which means that the relationship in Equation (9) is considered statistically significant at the confidence level greater than 99%. Standard errors of the coefficient of $\ln({\mathrm{Age}}_{\mathrm{conc}})$ and the intercept are 0.124 (*p*-value < 0.001) and 0.332 (*p*-value < 0.001), respectively. Therefore, the coefficient of RH and the intercept in Equation (9) are statistically significant at the confidence level greater than 99%. 

Given concrete age, Equations (8) and (9) result in very similar dielectric constant values, with a mean absolute error of 0.05. However, Group R4 (relative humidity greater than 70%) shows a slightly greater dielectric constant for the same ages compared to the other three groups with an approximate equation,

(10) $$\varepsilon_{r,c}^{\mathrm{Age},\mathrm{RH}>70\%}=-1.47\ln({\mathrm{Age}}_{\mathrm{conc}})+12.80\;\mathrm{for}\;2\leq {\mathrm{Age}}_{\mathrm{conc}}<43\;\mathrm{years}$$

F-statistic of the nonlinear regression model is 28.225 (*p*-value < 0.001), which means that the relationship in Equation (10) is considered statistically significant at the confidence level greater than 99%. Standard errors of the coefficient of $\ln({\mathrm{Age}}_{\mathrm{conc}})$ and the intercept are 0.26 (*p*-value< 0.001) and 0.90 (*p*-value < 0.001), respectively. These statistical analysis results indicate that the coefficient of $\ln({\mathrm{Age}}_{\mathrm{conc}})$ and the intercept are statistically significant at the confidence level greater than 99%.

### 4.3. A Practical Curve of Concrete Dielectric Constant in Asphalt-Covered Bridge Decks 

In this study, Equation (9), for the relative humidity less than or equal to 70%, is proposed to be a practical curve describing the age-related variation of the dielectric constant of concrete bridge decks with asphalt overlay on Korean Expressways for the 1 GHz air-coupled GPR system. A calibration factor is proposed to suppress the effect of relative humidity on the dielectric constant of concrete as follows,

(11) $$\gamma \left({\mathrm{Age}}_{\mathrm{conc}}\right)=\{\begin{array}{c} \;1\;\mathrm{for}\;\mathrm{RH}\leq 70\%\\ {\;\varepsilon }_{r,c}^{\mathrm{Age},\mathrm{RH}\leq 70\%}\left({\mathrm{Age}}_{\mathrm{conc}}\right)/\varepsilon_{r,c}^{\mathrm{Age},\mathrm{RH}>70\%}\left({\mathrm{Age}}_{\mathrm{conc}}\right)\;\mathrm{for}\;\mathrm{RH}>70\% \end{array}$$

**Figure 8:**
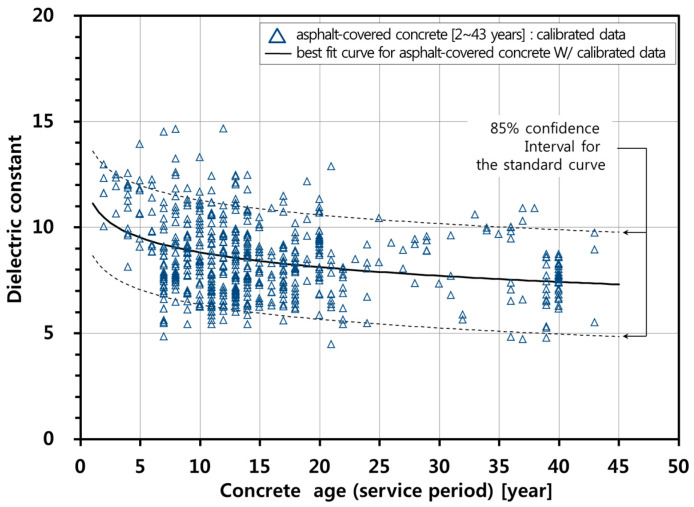
Proposed standard curve representing the relationship between the dielectric constant of concrete bridge deck and concrete age (service year).

Based on the field experiences and the data analyses in this study, a dual cut-off dielectric constant criterion is proposed to determine the severity of concrete deterioration in concrete bridge decks with asphalt overlay. Note that the upper and lower bounds were suggested as criteria to evaluate the deteriorated concrete in the relatively wet and dry conditions, respectively. The concrete can be interpreted as nearly sound (not significantly deteriorated) with a confidence level of (1 − α) when the relative permittivity of concrete in bridge deck is within the upper and the lower boundaries as follows [27],

(12) $$\varepsilon_{r,c}^{\prime }-t_{(1-\alpha /2),n-2}s_{\varepsilon |\ln(\mathrm{Age})}\leq \varepsilon_{r,c}^{\prime }\leq \varepsilon_{r,c}^{\prime }+t_{(1-\alpha /2),n-2}s_{\varepsilon |\ln(\mathrm{Age})}$$

where $\varepsilon_{r,c}^{}$ is the measured dielectric constant of asphalt-covered concrete after considering the effect of relative humidity (=$\varepsilon_{r,\mathrm{survey}}\times \gamma ({\mathrm{Age}}_{\mathrm{conc}})$, $\varepsilon_{r,\mathrm{survey}}$ is the measured dielectric constant of concrete using the 1 GHz air-coupled GPR system determined by using Equations (5) and (6)); $\varepsilon_{r,c}^{\prime }$ is the estimated dielectric constant of asphalt-covered concrete determined by Equation (9); *t*_(__1−*α*/2),*n*−2_ is the *t*-distributed variate at the probability of (1 − *α*/2) with (*n* − 2) degree-of-freedom; *n* is the number of samples (in this study *n* = 573); and $s_{\varepsilon |\ln(\mathrm{Age})}$ is the conditional standard deviation

(13) $$s^{2}{}_{\varepsilon |\ln(\mathrm{Age})}=\frac{\Delta^{2}}{n-2}=\frac{\sum_{i=1}^{n}(\varepsilon_{i}-{\varepsilon_{i}}^{\prime }{)}^{2}}{n-2}$$

where ε*_i_* is the measured value and ${\varepsilon_{i}}^{\prime }$ is the estimated value from the fitted line. For example, the dash lines in Figure 8 show the upper and lower boundaries at a confidence level of 85%. The confidence level of 85% was determined by taking into account several engineering factors (variability of concrete quality, construction error and uncertainties in GPR measurements and analyses) and government policy on the quality control and quality assurance. 

## 5. Application of the Proposed Model by a GPR Field Survey

### 5.1. Target Bridge for the Field Survey

**Figure 9:**
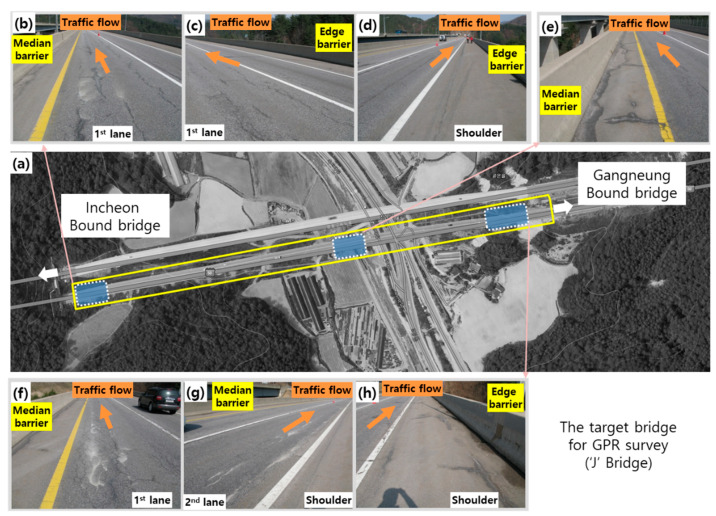
Overview of the target bridge (“J” bridge) for validation of the proposed GPR signal interpretation): (**a**) a satellite map of the target “J” bridge, (**b**–**h**) pictures presenting deterioration conditions on the surface of bridge decks in the target “J” bridge.

The target ‘J’ bridge is a steel box girder bridge constructed in 1999 with an extension of 600 m, a width of 12.15 m, and offers three traffic lanes including the road shoulder (Table 2, Figure 9). The concrete bridge deck in the ‘J’ bridge was overlaid with asphalt concrete. The target ‘J’ bridge is located at Pyeongchang on the Yeongdong expressway. Average temperature of the location is about −10 °C between December to March [25]. A great deal of deicing chlorides has been sprayed in this heavily snowed and cold region, which caused chloride-induced deterioration in concrete and created a corrosive environment of rebars in concrete. 

### 5.2. GPR Survey

**Figure 10:**
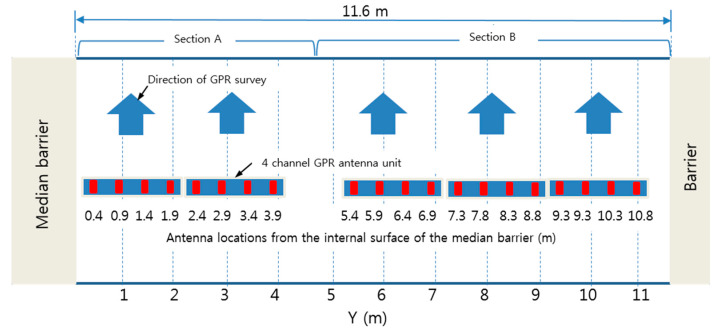
Illustration of GPR surveys on the target pilot ‘J’ bridge using a 1 GHz air-coupled GPR system (see Figure 2).

The test bridge has three lanes including a shoulder with a total width of 12.15 m and an effective width of 11.6 mm (distance between internal surfaces of barriers). A traffic lane was blocked during the survey time for a more accurate survey. To avoid traffic interruption, the bridge was divided into two sections (sections A and B in Figure 10). The GPR survey was performed on one section at a time, while blocking the other section. In the GPR survey of section A, GPR antennas were located at *y* = 0.4, 0.9, 1.4, 1.9, 2.4, 2.9, 3.4, 3.9 m: whereas for section B, GPR antennas were located at *y* = 5.4, 5.9, 6.4, 6.9, 7.3, 7.8, 8.3, 8.8, 9.3, 9.8, 10.3, 10.8 m (see Figure 10). Consequently, a total of 20 lines of GPR scanning were conducted with a resolution of 0.4–1.5 m (the center-to-center distance between antennas) in the transverse direction. The distance between the four antennas mounted on GPR vehicle is fixed at 0.5 m. A total of 144,848 scan data were collected in the manner of 12 scans/m in the direction of the bridge axis. 

The survey was performed on 8 April 2010, and the bridge was in service for 11 years. Therefore, the expected value of the dielectric constant was 8.7 for sound concrete bridge decks in accordance with Equation (9). The atmospheric relative humidity measured at this time was 51%, which was below the critical relative humidity value of 70%, requiring no calibration. In addition, the lower limit and upper limit of dielectric constant value based on Equation (12) and Figure 8 were 5.5 and 11.9, respectively (85% confidence level). The upper limit was close to 12, the threshold dielectric constant value of the single dielectric constant criterion for deterioration evaluation of asphalt-covered concrete bridge decks in Korean expressways [13,14]. The survey was performed in spring season, and it snowed or rained a little in the region of the survey for two weeks before the survey date (Table 3). On the survey day, the surface of the road was air-dried, without standing water or snow. In addition, the temperature during the GPR survey was in a range of 10 to 14 °C, which indicates the water in the air-void of asphalt and concrete was not frozen. 

**Figure 11:**
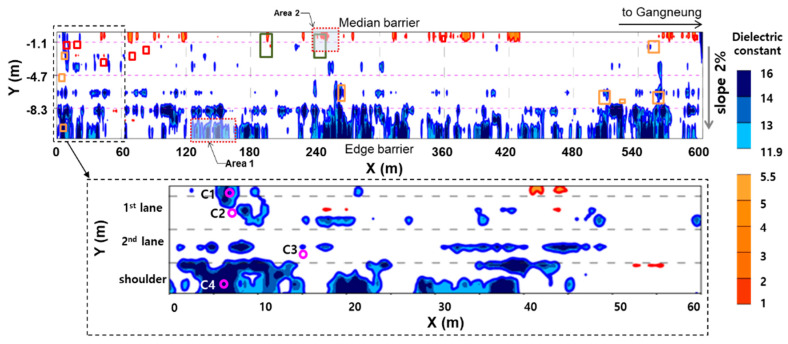
Plane view of dielectric constant of top concrete on the pilot target bridge deck: (red line-5.5, blue line-11.9). Surface deteriorations based on visual inspection are presented as: 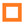 efflorescence, 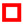 map cracking, 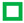 patching).

The average and standard deviation of measured dielectric constant of concrete were 9.6 and 2.4, respectively. The dielectric constant of concrete in the GPR field survey was calculated by Equations (5) and (6). The measured average was a little greater than that of the predicted value, 8.7. According to the field experiences from KEC, this discrepancy is attributed to water penetration into concrete of bridge decks from the rain before the survey date through the damaged area of pavement and deteriorated part of waterproofing [28]. Figure 11 shows the condition map of the concrete deck on the target pilot bridge based on the GPR survey. The horizontal and vertical axes of the deck in Figure 11 represent the extension of the bridge in the longitudinal direction and the distance from the median barrier in the transverse direction of the bridge, respectively. The origin of the vertical axis is located at the median barrier, with increasing value toward the road shoulder. Dielectric constant of concrete measured at the location of the bridge (*x,y*),$\;\varepsilon_{r,c}^{}\left(x,y\right)$, was calculated by Equations (5) and (6) using the amplitude values of the reflected waves from the air-asphalts surface (A1) and from the asphalt-concrete interface (A2) extracted from the GPR B-scan image at (*x,y*). Note that the *x* coordinate was read from the GPR B-scan image (see Figure 4) and the y coordinate was determined by measuring the distance of the antenna used for generating the GPR B-scan image from the median barrier. A grid of the condition map was defined to have 50 rows and 1200 columns with equal spaces in the *x* and *y* directions, respectively. Amplitude values on the grid system were obtained by interpolation of the measured data set (*x,y*,$\varepsilon_{r,c}^{}\left(x,y\right)$) using the kriging algorithm and visualized using a commercially available graphic program (Surfer^®^) [29]. 

In Figure 11, the regions with a dielectric constant value, the over upper cut-off value of 11.9 are shown as color blue, and the regions under the lower cut-off value of 5.5 are shown as color red. Overall, higher values (‘blue’ areas) of concrete dielectric constant were observed in the rightmost lane (i.e., close to edge barrier), while lower values (‘red’ areas) were observed in the leftmost lane (i.e., close to median barrier). This occurrence can be explained by the transversal slope of the pavement (i.e., 2% slope) that leads to migrating the surface water towards the right lane. Deteriorated concrete with enhanced porosity and/or dense microcracks would result in higher dielectric constant in the wet conditions (i.e., regions close to edge barrier) since the void system in the deteriorated concrete could be filled with water. On the contrary, the void caused by concrete deterioration could be filled with air, which would result in lower dielectric constant in the dry conditions (i.e., regions close to median barrier). Therefore, the test regions shown as blue and red colors can be interpreted as likely deteriorated concrete regions in the wet and dry conditions, respectively. For comparison, surface deteriorations based on visual inspection are presented as: 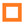 efflorescence, 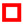 map cracking, 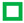 patching, in the same figure. The part of bridge decks where efflorescence was observed (marked with ‘
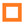
’) exhibited high dielectric constant values. It is expected that continuous water penetration and deterioration of the inside is developing there. One interesting finding is that some damaged (or solid) regions determined from concrete dielectric constant were interpreted as solid (or damaged) regions by visual inspection on the surface. For example, the deteriorated areas according to the dielectric constant are mostly located near the median barrier and shoulder, where no obvious deterioration is observed by visual inspection on the surface. In contrast, the dielectric constant values in most of the traffic lanes are between the cut-off values, while there are many surface-breaking cracks in the traffic lanes.

**Figure 12:**
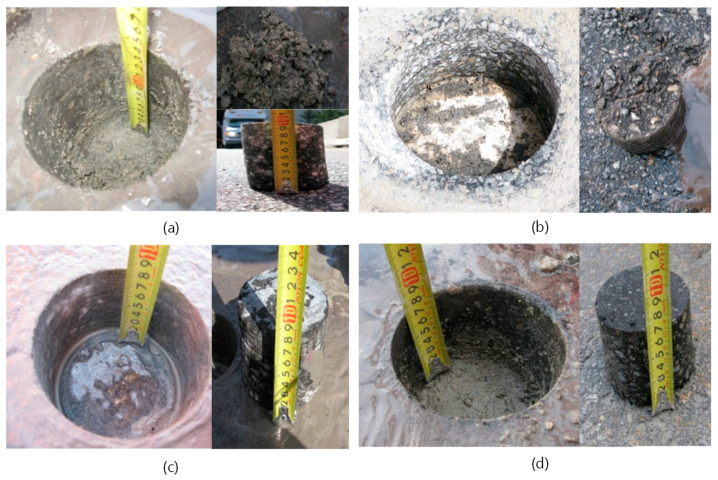
Evaluation of condition of asphalt-covered concrete bridge decks through core extraction from the four locations in the target bridge (“J” bridge): (**a**) core 1 (C1), (**b**) core 2 (C2), (**c**) core 3 (C3), and (**d**) core 4 (C4) (see Figure 12). Depth of deterioration in concrete decks and the condition of water-proofing-layer in the A/C interface were evaluated.

Core samples were extracted from four locations near the shoulder and the median barrier to examine the actual condition of concrete decks under asphalt overlay (see Figure 12 and Table 4). Two cores of C1 and C4 were extracted from the regions with the dielectric constant over 11.9, while two cores of C2 and C3 were from the regions with the dielectric constant within the two cut-off values. In the two cores of C2 and C3, the concrete and asphalt overlay have good bonding, and there was no evidence of concrete deterioration. However, it was observed that the waterproofing layers of the two cores C1 and C4 were deteriorated, and the interface between concrete and asphalts overlay was completely debonded. The deteriorated depths of concrete in the cores C1 and C4 were 35 and 10 mm, respectively. It was reported that weak bonding between the asphalt overlay and concrete deck can be a sign of deterioration of concrete [28]. The rainwater and deicing salt penetrated the area of the damaged overlay, and then penetrated the concrete through the weak points with poor waterproofing. Subsequently, the freeze-thaw action could result in enhanced porosity through weakening and/or softening of the microstructure. 

**Figure 13:**
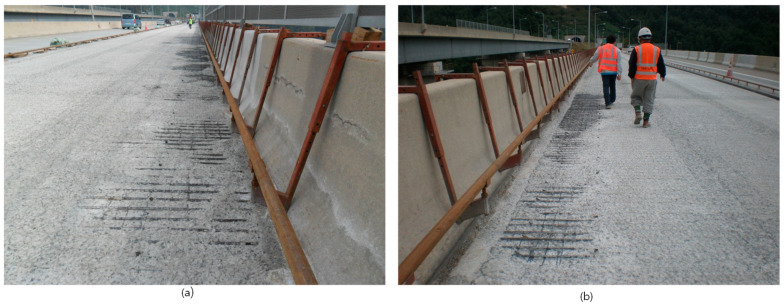
Actual concrete condition after removing the asphalt overlay and deteriorated concrete using a high-pressure water-jet machine in the target bridge (“J” bridge) after the GPR survey: (**a**) area 1 and (**b**) area 2 (see Figure 11).

In addition, the actual conditions of larger areas of concrete bridge decks under asphalt overlay were examined by visual inspection after removing the asphalt overlay and the deteriorated parts of concrete using high pressure water jetting. Figure 13a,b is pictures presenting concrete decks in the target pilot bridge after the water jetting process. Figure 13a clearly shows that the region with higher dielectric constant than the upper limit exhibits severely deteriorated concrete near the shoulder (area 1) although there were no visually observed damages on the surface. The deteriorated depth in the shoulders, with dielectric constant over 12, was deeper than the depth of rebars in the upper layer. Furthermore, Figure 13b shows that the region with a lower dielectric constant than the lower limit exhibits concrete deterioration deeper than rebars. It can be demonstrated that deteriorated concrete with enhanced porosity and/or dense microcracks can be identified by a higher or lower dielectric constant in the wet conditions or dry conditions, respectively. Note that the deteriorated concrete in the dry conditions could be overlooked when using visual inspection or GPR signal interpretation based on the conventional single cut-off dielectric constant criteria. The deteriorated area and ratio of reduction based on the single value criteria were 936 m^2^ and 13%, respectively. The dual cut-off criterion resulted in 1038 m^2^ (51 m^2^ + 987 m^2^) and 15%, respectively. Consequently, these results demonstrated two important conclusions in this study: (1) the practical GPR interpretation method based on concrete dielectric constant is effective for evaluating the condition of concrete under asphalt overlay, which could not be directly estimated by visual inspection on the surface and (2) the double cut-off criteria for dielectric constant could further improve the capability of the practical GPR interpretation method in asphalt-covered concrete bridge decks in the relatively dry conditions, which could be not effectively estimated by the conventional single cut-off dielectric constant criterion. 

## 6. Conclusions

This study carried out GPR tests of asphalt-covered bridge decks currently in service to investigate the age-related variations of the dielectric constant of concrete in asphalt-covered bridges on Korean expressways. As a result, a standard curve of the dielectric constant of top concrete of asphalt-covered bridge decks with mid-to long-term service age was established. The following conclusions are derived in this study.

(1) The influence of atmospheric relative humidity (or seasonal variation) on asphalt-covered concrete bridge decks of 2–43 years of service performance was investigated. As relative humidity changed to 10%, the dielectric constant of concrete changed to about 0.11. This is about 12% of the bare concrete bridge deck, indicating that the influence of relative humidity (air) is blocked by asphalt overlay and a waterproof layer on the concrete bridge deck.
(2) Dielectric constant of concrete was found to be influenced by the service age of the bridges in most expressway routes based on the observed values of the dielectric constant of concrete in the bridges with 2–43 years of service. This pattern was similar to the bare concrete bridge decks. This study proposed a practical curve describing the variation of the dielectric constant of the top concrete of bridge decks with asphalts overlay in consideration of service life with a confidence level of 85%. Based on the proposed model and field survey experiences on Korean expressways, a double cut-off dielectric constant criterion was newly proposed for condition assessment of asphalt-covered concrete bridge decks on Korean expressways.
(3) A case study of GPR application at an actual bridge on the Yeongdong expressway in Korea demonstrated that the proposed GPR signal interpretation method, based on the double cut-off dielectric constant criterion, is useful for better interpretation of concrete dielectrics and beneficial for reliable condition assessment of asphalt-covered concrete bridge decks in Korean expressways. However, the validation was obtained from only a single target bridge in Korea. Therefore, more studies are still needed to verify the validity of the practical curve and the methodology described in this study by systematic comparison researches using the data from other bridges in the field.

## Figures and Tables

**Table 1:** Details of survey bridges in this study.

Route (Expressway)	Bridge Length (m)	No. of Lanes	Survey Year	No. of Bridges	No. of Survey Times	Completion Year
Gyeongbu	8–505	1–6	’02–’13	137	157	’69–’70, ’86–’88, ’91–’93, ’95–’96, ’98–’99, ’02,’03, ’06
Yeongdong	16–780	1–4	’04–’13	102	118	’71, ’76, ’91, ’94, ’97, ’99, ’00, ’01, ’06
Jungbu	16–750	1–3	’06–’13	92	101	’87, ’98–’01, ’05
Honam	10–603	1–6	’99, ’04–’06, ’09–’13	74	87	’70, ’73–’74, ’85–’86, ’89, ’92–’93, ’96, ’98–’99, ’02, ’12
Jungang	11–830	1–3	’02–’12	64	82	’95–’96, ’99, ’00–’01
Seohaean	15–710	1–5	’08–’13	56	58	’94, ’96–’98, ’00–’02
Seoul outer ring	30–1219	1–5	’06, ’08–’10, ’12, ’13	31	36	’91–’93, ’95, ’98–’99, ’02
Namhae	25–331	2–4	’03, ’10–’13	16	16	’73, ’81, ’83, ’91–’93
Jungbunaeryuk ^(1)^	10–300	2, 5	’09–’12	12	12	’78, ’84, ’95, ’02, ’04
Daejeong south	29–505	2, 3	’08, ’10	8	8	’00
Pyeongtaek- Jecheon	24–115	3	’12	4	4	’02
88 ^(2)^	103, 206	2	’05, ’06	2	2	’84
Donghae	125	2	’09	1	1	’01
Ulsan	175	4	’04	1	1	’69
Iksan- Pohang	480	2	’12	1	1	’01
Subtotal	8–1219	1–6	’99–’13	601	684	

^(1)^ Part of the existing route was renamed and integrated. ^(2)^ Old route was closed and new route with the same name ‘88’ was constructed.

**Table 2:** General information of the pilot target bridge (“J” bridge) for field survey.

Route	Bridge Name	Super-Structure	Completion Year	Location	Length ^(2)^	No. of Lanes	Survey Year
Youngdong expressway	J	SBG ^(1)^	1999	Pyeongchang	10@60 m = 600 m	3	2010

^(1)^ Steel Box Girder Bridge; ^(2)^ the number of spans = 10, the length of each span = 60 m.

**Table 3:** Weather conditions of the pilot target bridge J for 14 days before the survey day ^(1)^.

Survey Date	Relative Humidity	Season/Weather	Remarks	
**8 April 2010**	51%	Spring/Brume	[Daily temperature] average: −0.7–−3.0 °C highest: −0.9–23.7 °C lowest: −9.5–4.5 °C	[Precipitation] amounts: 0–7.7 mm (rain or snow) days: 9 days note: fog 8 days

^(1)^ Weather data from Korea Meteorological Administration (KMA) includes rain sensing days.

**Table 4:** Investigation result of core samples (1st span).

Classification	Location	Deteriorated Depth (mm)	Dielectric Constant	Remark
C1	Near median	35	13	Debonding
C2	1st lane	-	10	Bonding
C3	2nd lane	-	8	Bonding
C4	Shoulder	10	17	Debonding
